# Spatial divergence between self-rated health and body mass index in China: Exploring the role of economic status using China Family Panel Studies (CFPS) 2016 and 2020

**DOI:** 10.1371/journal.pone.0339123

**Published:** 2025-12-31

**Authors:** Jiayin Liu, Peizhi Yu, Xiaoyu Ye, Yirui Yang, Zhixin Feng

**Affiliations:** 1 School of Geography and Planning, Sun Yat-Sen University, Guangzhou, China; 2 Guangdong Key Laboratory for Urbanization and Geo‑simulation, Sun Yat-Sen University, Guangzhou, China; 3 Guangdong Provincial Engineering Research Center for Public Security and Disaster, Guangzhou, China; Southwestern University of Finance and Economics, CHINA

## Abstract

Health disparities across China remained a major public health concern, with both individual and regional economic conditions shaping variations in health outcomes. In the context of the “Healthy China 2030” initiative, this study used data from the 2016 and 2020 waves of the China Family Panel Studies (CFPS) and applied multivariate multilevel logistic regression models to examine the associations between economic status at the individual and province levels and two key health indicators: self-rated health (SRH) and body mass index (BMI). The results showed that, at the individual level, a higher total value of durable consumer goods was negatively associated with having a normal BMI, whereas higher travel expenditure, per capita living space, and total cash and deposits were associated with a greater likelihood of reporting good SRH. At the province level, higher mean income exhibited a negative association with normal BMI and moderated the effects of household economic resources: income growth reduced health disparities in some dimensions (e.g., living space, cultural and entertainment spending) but widened them in others (e.g., vehicle purchase expenses). Spatial mapping further indicated that southern provinces tended to exhibit healthier BMI distributions but lower levels of good SRH compared with northern regions, revealing a clear spatial divergence between subjective and objective health measures. These findings highlighted the complex, multilevel, and spatially uneven relationships between economic status and health in China. Policy efforts should aim to strengthen household economic resilience, reduce regional health inequalities, and tailor interventions to local contexts to promote health equity nationwide.

## 1. Introduction

Health is a fundamental component of national prosperity and societal well-being. Over the past four decades, China’s rapid economic growth has profoundly reshaped social structures, living conditions, and health outcomes, simultaneously improving overall health while widening disparities across regions and population groups. As socioeconomic development accelerates and living standards rise, both policymakers and the public increasingly recognize the importance of individual health. The “Healthy China 2030” initiative underscores not only the goal of improving population health but also the imperative to reduce health inequalities, highlighting the need to understand how different dimensions of economic status shape health.

Economic status at different levels influences residents’ health in distinct ways. At the province level, mean income reflects the overall degree of regional development, including the availability of public resources, healthcare infrastructure, and social welfare, which collectively shape population health outcomes. Evidence indicates that higher-income regions typically provide better healthcare, higher education levels, improved living environments, and healthier lifestyles, thereby enhancing overall population health [1]. In contrast, lower-income regions often face poorer health conditions, higher disease prevalence, and reduced life expectancy [2].

At the same time, economic development also introduces new health challenges. Although economically advanced provinces are characterized by stronger health systems, richer health promotion resources, and greater health investments [3], rapid economic expansion can widen income gaps and exacerbate inequalities in living standards and healthcare access. In addition, once economic prosperity reaches a certain threshold, the marginal health returns may plateau or even reverse due to environmental pollution, lifestyle-related chronic diseases, and psychological stress associated with affluent societies. These dynamics are clearly reflected in China’s pronounced north–south health divide [4,5]. Consequently, interprovincial differences in economic development shape both health behaviors and outcomes, underscoring the need to consider macroeconomic conditions when assessing health inequalities.

At the individual and household levels, economic status is equally critical, influencing access to medical services, lifestyle patterns, and living environments, factors that affect both objective and subjective health perceptions. Early research established that higher income improves individual health [6,7]. Recent studies indicate that income inequality affects both personal and community health and is associated with population mortality [8–13]. Household economic conditions, encompassing income, expenditures, assets, savings, and investments, provide a comprehensive measure of financial stability that directly impacts health [14]. Wealthier individuals typically enjoy better healthcare access, healthier lifestyles, and more favorable health outcomes, whereas low-income households face financial stress, poor living conditions, and environmental risks that negatively affect health [15–17].

Economic resources further influence health through household expenditures on healthcare, food, transportation, culture, and travel. Adequate healthcare spending ensures timely access to essential services, although excessive medical costs may generate financial hardship. Food expenditures influence diet quality and health outcomes, while lifestyle-related spending, including travel and cultural activities, promotes both physical and mental well-being [18,19]. Yet economic advantage may also entail health risks: high-income groups are more likely to experience overnutrition, sedentary lifestyles, and obesity-related health problems [12,20–23].

Although substantial research has examined the influence of individual and household economic status on health, relatively less attention has been paid to the moderating role of regional economic development. In economically advanced provinces, well-developed healthcare systems and strong public health awareness can narrow socioeconomic health disparities, benefiting even low-income residents. Conversely, in regions where rapid economic development heightens social stratification and lifestyle differences, health disparities may widen, with affluent groups enjoying disproportionate health advantages. These patterns highlight the importance of assessing how provincial economic contexts shape the micro-level relationship between economic resources and health.

Despite extensive research, the extent to which province development shapes the link between personal economic resources and health outcomes remains insufficiently understood, leaving important gaps in explaining how socioeconomic and spatial inequalities in health emerge and persist across China. This study addresses these gaps by examining the association between economic status and two key health outcomes, Body Mass Index (BMI) and Self-Rated Health (SRH). Body Mass Index (BMI) and self-rated health (SRH) are widely used as complementary health indicators. BMI objectively reflects body weight status and is closely associated with the risk of chronic diseases such as diabetes and cardiovascular conditions, as well as premature mortality [24]. In contrast, SRH captures individuals’ subjective perceptions of their overall health and has demonstrated strong predictive validity for morbidity and mortality [25,26]. Importantly, the relationship between BMI and SRH varies across contexts, influenced by weight-change patterns, body weight perceptions, and life satisfaction. Investigating these outcomes jointly across diverse province contexts thus provides critical insights into China’s evolving health landscape.

### 1.1. Theoretical mechanism and research hypothesis

The relationship between economic status and health can be better understood through established theoretical perspectives. From both macro- and micro-level dimensions, two classical theories, the absolute income theory and the health capital theory, collectively explain the pathways through which economic resources shape health. The absolute income theory posits that an individual’s or region’s overall economic level directly influences health outcomes by determining access to healthcare, nutrition, housing, education, and other essential social determinants of health. In essence, as economic resources increase, individuals and communities are better able to afford health-promoting goods and services, leading to overall improvements in population health [27,28]. The health capital theory conceptualizes health as a form of capital in which individuals and households invest through time, money, and behavioral choices to maintain or enhance their physical and mental well-being. According to these theories, economic resources determine the ability to invest in preventive care, nutritious diets, and healthier environments, while the effectiveness of these investments depends on individual characteristics and broader socioeconomic conditions [6,20,29,30].

Drawing on these theoretical perspectives, this study proposes three hypotheses:

**H1**: Higher individual and household economic status is associated with better self-rated health (SRH), but also with a higher likelihood of abnormal BMI, reflecting health risks linked to affluence, such as overnutrition and sedentary lifestyles.

**H2**: The association between household economic resources and health outcomes (BMI and SRH) is moderated by provincial economic development. In economically advanced provinces, expenditures on food, healthcare, travel, and cultural activities have stronger positive effects due to better infrastructure and service availability. In less developed provinces, similar expenditures yield weaker health benefits.

**H3**: Geographic patterns in BMI and SRH persist even after accounting for individual, household, and provincial economic status, indicating spatial clustering of health outcomes beyond economic explanations.

By employing multivariate multilevel logistic regression models, this study provides new empirical evidence on how different dimensions of economic status, across individuals, households, and provinces, shape both objective and subjective health outcomes. The findings contribute to a more comprehensive understanding of health inequalities in China and offer policy-relevant insights that support the goals of “Healthy China 2030.”

## 2. Data and methods

### 2.1. Data

This research used datasets from two waves of the China Family Panel Studies (CFPS), specifically, the 2016 wave and the 2020 wave, coinciding with the year China launched the “Healthy China 2030” Planning Outline and initiative, respectively. CFPS is a national representative, survey, with a sampling frame that included 31 province administrative regions across Mainland China，excluding Macau, Hong Kong, and Taiwan. Employing a multistage probability sampling method, the CFPS is designed to collect longitudinal data at the individual, household, and community levels within contemporary China. The final analytic sample consisted of 27,510 adults aged 18 and above in 2016, and 20,982 adults aged 18 and above in 2020. Given that the CFPS provides province names, province level information for 2016 and 2020 were sourced from the 2023 China Statistical Yearbook. The dataset features a hierarchical structure, with individuals nested within repeated years (waves) and provinces.

### 2.2. Dependent variables

The CFPS datasets facilitate health assessments through two primary measures: Body Mass Index (BMI) and self-rated health (SRH). BMI is a widely accepted metric for evaluating an individual’s weight status, a simple numerical measure based on height and weight [31]. It serves to identify weight categories that may predispose individuals to health issues, noting that both abnormally high and low BMI levels can signal potential health risks. For instance, obesity is associated with cardiovascular diseases, while a low BMI may indicate problems with the immune system [32]. According to the China’s National Physical Fitness Measurement Standards, a BMI range of 18.5-24.9 is considered normal (coded as 1), while values outside this spectrum are deemed to reflect an unhealthy weight status (coded as 0).

Self-rated health is a subjective health measure which allows respondents to subjectively evaluate their own health [33]. In the CFPS, self-rated health is categorized into five levels: “excellent”, “very good”, “good”, “fair”, and “poor”. For analytical purposes, the categories “excellent”, “very good” and “good” are grouped together to indicate good health (coded as 1), while “fair” and “poor” are combined to represent poor health (coded as 0). These indicators are essential for understanding the health conditions of the studied population.

### 2.3. Independent variables

The key independent economic variables in this study are categorized into two levels: macro and micro. At the macro level (province), average income serves to reflect the economic environment in which the respondents reside. At the micro level (individual or household), ten variables are considered: total income from employment, total government subsidies, monthly food expenses [34], cultural and entertainment expenses, travel expenses, car purchase expenses, healthcare expenses [35], per capita living areas [36], total value of durable consumer goods, and total cash and deposits. In this study, per capita living area (PCLS) is defined as the total residential floor area of a household divided by the number of its members. Apart from PCLS, all economic variables are log-transformed for analysis. These variables encompass three dimensions of the individual/household economy: cash and savings, expenditure, and social class. To address potential multicollinearity within the model, a Variance Inflation Factor (VIF) test was performed, confirming that all independent variables displayed VIF values within acceptable ranges.

This study incorporates several control variables to account for potential confounding factors: age [37], gender [38], hukou (household registration), and highest level of education attained [39]. In addition, for respondents who also participated in earlier survey waves, their BMI and self-rated health (SRH) from 2014 and 2018 were included as covariates to adjust for prior health status and enhance temporal comparability across waves.

### 2.4. Analytical approach

Given the two health outcomes and the hierarchical structure of the data, a multivariate multilevel model is the appropriate analytical approach [40]. This statistical method allows for the simultaneous examination of multiple dependent variables while accounting for the nested nature of the data. In this study, Body Mass Index (BMI) and self-rated health (SRH) are analyzed together rather than separately, providing a more comprehensive understanding of their shared and distinct associations with common predictors. These two health outcomes served as the Level-1 response variables in the multivariate multilevel model. Additionally, this model enables the assessment of the correlation between BMI and SRH after controlling for all independent variables, ensuring more robust and context-sensitive findings. The analysis unfolds through a series of four models, each progressively examining the influence of various factors on health outcomes. Model 1 includes seven control variables: age, gender, hukou status, highest academic qualifications, and BMI and SRH statuses from 2014 and 2018 (entered respectively in the BMI and SRH models). Model 2 expands this framework by incorporating average income at the province level. Model 3 introduces ten economic variables at the individual and household levels, alongside individual health status. Model 4 further explores cross-level interactions between province-level average income and the ten economic variables at the individual and household levels. All models are computed using MLwiN version 3.13 [40].

## 3. Results

### 3.1. Descriptive analysis

Table 1 presents the dependent and independent variables across the two survey waves. Notably, approximately 64% of respondents had a normal BMI in 2016, but this proportion declined to 54% by 2020. In contrast, the percentage of respondents reporting good self-rated health (SRH) increased from 67% in 2016 to 76% in 2020. Regarding economic status at both the individual and province levels, all indicators exhibited an upward trend between 2016 and 2020. These patterns suggest that changes in economic status may be differently associated with BMI and SRH.

**Table 1:** Descriptive univariate information for individual and province variables.

		*2016*	*2020*	*Total*
**Dependent variables**	*N = 27510*		*N = 20982*	*N = 48492*
**BMI**				
Normal	63.93%		54.00%	59.63%
Abnormal	36.07%		46.00%	40.37%
**Self-rated Health**				
Good	67.41%		75.85%	71.06%
Others	32.59%		24.15%	28.94%
**Control variables**				
**Age**	In years: 18–98 Mean = 46.1		In years: 18–95 Mean = 45.87	18-98 Mean = 46.02
**Gender**				
Male	50.08%		50.43%	50.23%
Female	49.92%		49.57%	49.77%
**Hukou**				
Urban	28.92%		29.50%	29.17%
Rural	71.00%		55.85%	64.44%
Unknown	0.08%		14.66%	6.39%
**Highest academic qualifications**				
Primary	20.31%		17.11%	18.92%
Middle	27.35%		30.15%	28.57%
High school and above	15.07%		19.08%	16.8%
College and above	10.49%		17.04%	13.33%
**BMI in previous waves**				
Normal	43.24%		43.59%	43.39%
Abnormal	31.03%		38.05%	34.06%
New in this wave	20.35%		17.73%	19.22%
Missing	5.38%		0.63%	3.33%
**Self-rated health in previous wave**				
Good	51.44%		59.55%	54.95%
Others	28.21%		22.09%	25.56%
New in this wave	20.35%		17.73%	19.22%
Missing	0%		0.63%	0.27%
**Independent variables**				
***Economic status at the provincial level***				
**Mean income (10,000 RMB)**	1.36–5.43		2.03–7.22	2.71
***Economic status at the individual or household level***				
**Total income (10,000 RMB)**	0–220		0–600	3.04
**Total government subsidies (10,000 RMB)**	0–30		0–60.16	0.0912
**Monthly food expenses (10,000 RMB)**	0–7		0–10	0.0174
**Cultural and entertainment expenses (10,000 RMB)**	0–2		0–3	0.0289
**Travel expenses (10,000 RMB)**	0–15		0–11	0.1186
**Car purchase expenses (10,000 RMB)**	0–60		0–380	0.5603
**Healthcare expenses (10,000 RMB)**	0–200		0–16	0.0435
**Per capita living area (m**^**2**^)	0–800		0–430	6.7628
**Total durable goods value (10,000 RMB)**	0–600		0–4550000	25287
**Total cash and deposits (10,000 RMB)**	0–400		0–600	5.8548

### 3.2. Multivariate multilevel logistic regression results

Table 2 presents the results from multivariate multilevel logistic regression models analyzing the likelihood of having a normal BMI and good SRH. Model 1 reveals a negative association between age and both outcomes, indicating that as age increases, the likelihood of maintaining a normal BMI or perceiving one’s health as good declines (ORs = 0.996 and 0.968, respectively). Males were less likely than females to report a normal BMI (OR = 0.936) but more likely to perceive their health as good (OR = 1.319). Individuals with a rural hukou or without a defined household registration were more likely to report a normal BMI than those with an urban hukou (ORs = 1.047 and 1.101, respectively). However, those without a defined status were significantly less likely to report good SRH (OR = 0.866). Education also played an important role: individuals with at least a middle school education were more likely to report a normal BMI and perceive their health as good than those without formal education (ORs for normal BMI = 1.073, 1.101, 1.125; ORs for good SRH = 1.319, 1.640, 1.916, respectively). Among respondents interviewed in previous waves, those who had previously maintained a normal BMI were significantly more likely to continue doing so (OR = 7.338). Similarly, those who had reported good SRH in earlier waves were more likely to continue reporting good SRH (OR = 4.604).

**Table 2:** Multivariate multilevel model results for BMI and self-rated health (odds ratios with 95% confidence intervals).

	Model 1				Model 2				Model 3			
	Normal BMI		Good SRH		Normal BMI		Good SRH		Normal BMI		Good SRH	
	ORs	95% Cl	ORs	95% Cl	ORs	95% Cl	ORs	95% Cl	ORs	95% Cl	ORs	95% Cl
**Fixed Part**												
Age	0.996	(0.995–0.998)***	0.968	(0.966–0.969)***	0.997	(0.995–0.998)***	0.968	(0.966–0.969)***	0.997	(0.995–0.998)***	0.968	(0.966–0.969)***
Male (ref: female)	0.936	(0.899–0.975)***	1.319	(1.261–1.38)***	0.936	(0.899–0.974)***	1.319	(1.261–1.38)***	0.934	(0.898–0.973)***	1.323	(1.265–1.384)***
Hukou (ref: urban hukou)												
Rural hukou	1.047	(0.997–1.101)*	1.04	(0.983–1.1)	1.045	(0.994–1.097)*	1.041	(0.984–1.101)	1.036	(0.984–1.09)	1.074	(1.013–1.138)**
Unknown	1.101	(1.002–1.209)**	0.866	(0.781–0.96)***	1.1	(1–1.208)**	0.867	(0.782–0.961)***	1.089	(0.989–1.197)*	0.9	(0.811–1)**
Education (ref: No formal educated)												
Primary	1.034	(0.969–1.104)	1.053	(0.986–1.125)	1.035	(0.969–1.105)	1.053	(0.986–1.125)	1.037	(0.97–1.107)	1.046	(0.979–1.117)
Middle	1.073	(1.006–1.143)**	1.319	(1.235–1.411)***	1.074	(1.007–1.146)**	1.319	(1.235–1.409)***	1.08	(1.013–1.153)**	1.302	(1.218–1.391)***
High school	1.101	(1.021–1.186)**	1.64	(1.511–1.782)***	1.104	(1.023–1.19)***	1.639	(1.51–1.779)***	1.114	(1.034–1.202)***	1.602	(1.474–1.738)***
College and above	1.125	(1.034–1.226)***	1.916	(1.73–2.123)***	1.131	(1.038–1.231)***	1.91	(1.725–2.117)***	1.151	(1.054–1.257)***	1.788	(1.608–1.986)***
Missing	1.234	(1.063–1.432)***	1.09	(0.921–1.288)	1.235	(1.063–1.435)***	1.089	(0.922–1.287)	1.245	(1.071–1.448)***	1.07	(0.906–1.265)
BMI in the last wave (ref: Abnormal)												
Normal	7.338	(7.001–7.683)***			7.36	(7.022–7.706)***			7.374	(7.036–7.721)***		
New respondents	2.933	(2.773–3.105)***			2.939	(2.776–3.108)***			2.933	(2.773–3.105)***		
Missing information in the last wave	2.866	(2.575–3.19)***			2.866	(2.575–3.193)***			2.863	(2.573–3.19)***		
Self-rated heath in the last wave (ref: others)												
Good			4.604	(4.38–4.84)***			4.6	(4.375–4.836)***			4.586	(4.362–4.821)***
New respondents			3.068	(2.861–3.287)***			3.065	(2.861–3.287)***			3.056	(2.849–3.274)***
Missing information in the last wave			2.502	(1.733–3.615)***			2.507	(1.737–3.622)***			2.499	(1.732–3.611)***
Mean income at province level					0.929	(0.882–0.976)***	1.081	(1.01–1.157)**	0.937	(0.892–0.985)**	1.06	(0.988–1.137)
Total income from employment									1.000	(0.997–1.003)	1.003	(0.998–1.008)
Total government subsidies									0.986	(0.949–1.024)	0.969	(0.928–1.011)
Monthly food expenses									0.925	(0.825–1.037)	1.026	(0.898–1.174)
Expenses on culture and entertainment									1.1	(0.858–1.409)	1.117	(0.812–1.539)
Travel expenses									0.984	(0.939–1.03)	1.068	(1.007–1.134)**
Expenses on car purchases									0.999	(0.994–1.003)	0.997	(0.992–1.002)
Healthcare expenses									1.034	(0.962–1.112)	0.992	(0.914–1.078)
Per capita living area									1.001	(1–1.002)	1.002	(1–1.003)***
Total value of durable consumer goods									0.996	(0.994–0.999)***	1.000	(0.997–1.003)
Total cash and deposits									0.999	(0.998–1.001)	1.004	(1.002–1.006)***
Random Part												
Level4: province												
Variance	0.004		0.051		0.004		0.053		0.002		0.053	
Correlation	−0.007				−0.007				−0.008			
Level3: Year												
Variance	0.009		0.002		0.009		0.002		0.008		0.003	
Correlation	0.001				0.001				0			
Level2: individual (48492)												
Correlation	0.018				0.018				0.018			

Notes: ***p < 0.01, **p < 0.05, *p < 0.1.

Model 2, which incorporated mean province income, did not substantially alter the coefficients of other control variables. However, province mean income itself was significantly associated with both BMI and SRH, negatively with BMI and positively with SRH (ORs = 0.929 and 1.081, respectively).

To assess the relative importance of economic disparities at different levels, Model 3 incorporated both individual- and province-level economic indicators. The results show that among the ten individual or household economic variables, four exhibited significant associations with BMI or SRH, partially supporting Hypothesis 1, that higher individual and household economic status significantly influence personal health outcomes. However, not all indicators followed a straightforward pattern in which greater economic resources corresponded to better health. The total value of durable consumer goods was significantly associated with abnormal BMI, indicating that higher values of durable goods were linked to a decreased likelihood of maintaining a normal weight status (OR = 0.996). Significant positive associations were observed between SRH and tourism expenditure, per capita living area (PCLS), and total cash and deposits (ORs = 1.068, 1.002, and 1.004, respectively), suggesting that higher tourism expenditures, larger living areas, and greater household cash liquidity were associated with better self-rated health. These findings partially support H1, which proposed that higher individual and household economic status would be associated with better self-rated health (SRH) but might also increase the likelihood of abnormal BMI, reflecting potential health risks associated with affluence, such as overnutrition and sedentary lifestyles. By contrast, province mean income showed a negative association with BMI (OR = 0.937, p < 0.01) and was not significantly related to SRH. The variance components further indicate that inter-province differences accounted for only a small portion of total health disparities (BMI = 0.002; SRH = 0.053). In comparison, individual-level economic variables exhibited more consistent associations with both BMI and SRH. These results suggest that health outcomes in China are predominantly shaped by individual and household economic conditions, whereas province-level economic status contributes relatively little to observed variations. This underscores the greater importance of micro-level economic differences over macro-level province disparities in determining personal health.

**Fig 1:**
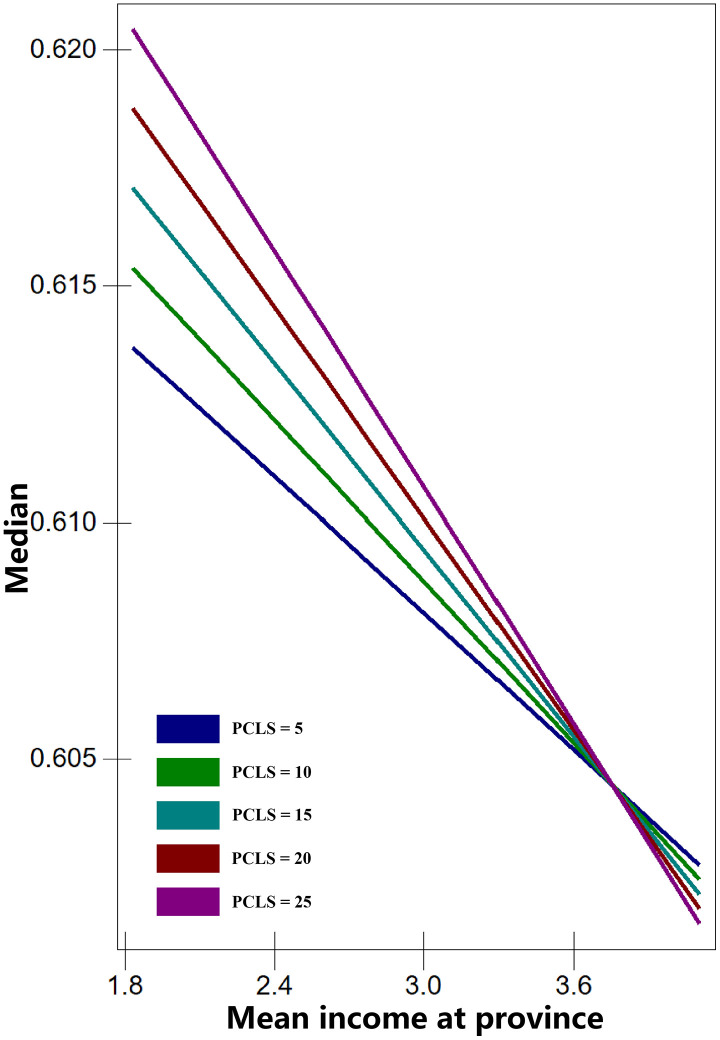
Predicted median BMI across all levels of per capita living area (PCLS).

**Fig 2:**
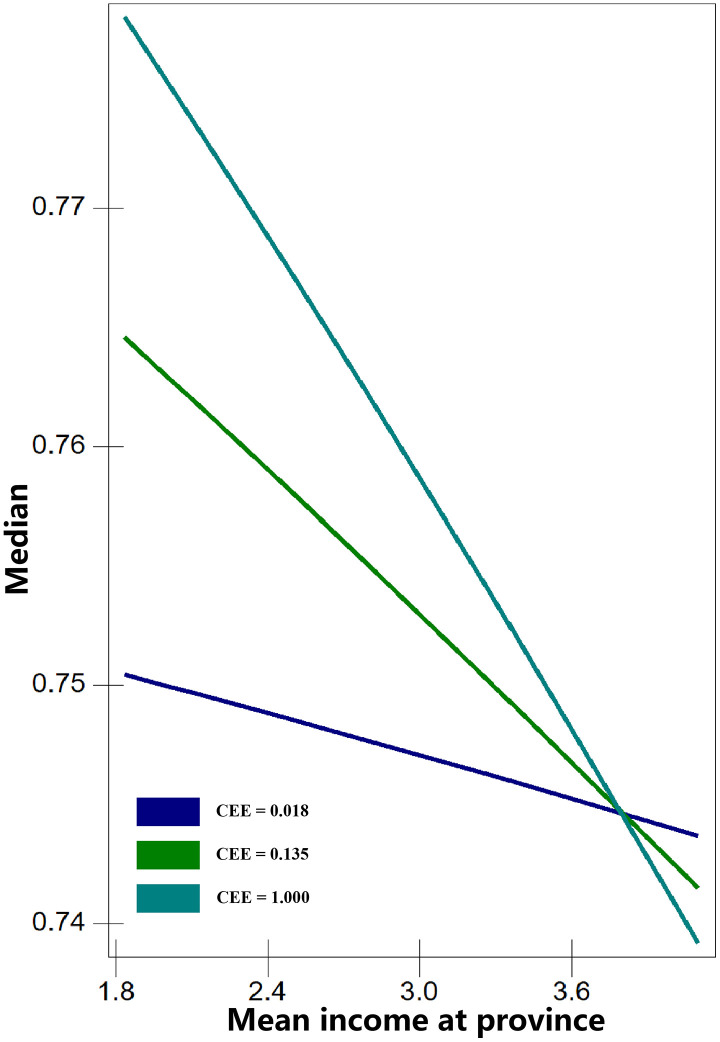
Predicted median SRH across all levels of cultural entertainment expenses (CEE).

**Fig 3:**
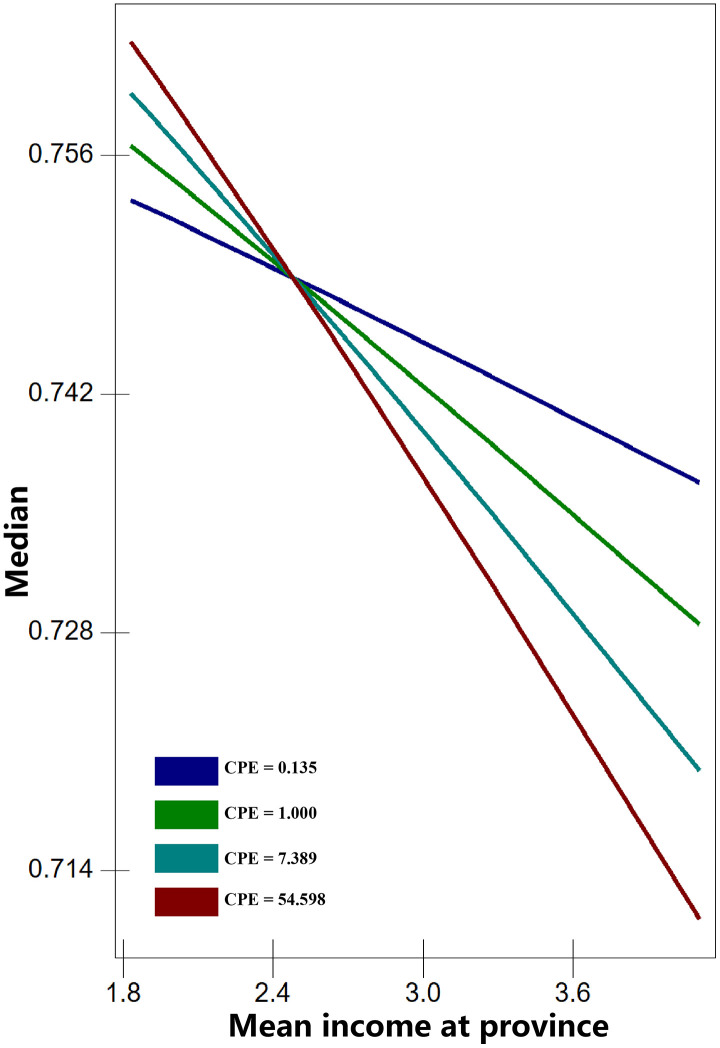
Predicted median SRH across all levels of car purchase expenses (CPE).

The results of Model 4 reveal significant cross-level interactions between province-level mean income and individual or household economic characteristics (interactions without significant associations are not shown). These findings support Hypothesis 2, indicating that province economic development selectively moderates the relationship between certain household economic resources and health outcomes. Specifically, one significant interaction was observed between per capita living area (PCLS) and province mean income for BMI (Model 4a). For self-rated health (SRH), two significant interactions were found: cultural entertainment expenses (CEE) × province mean income (Model 4b) and car purchase expenses (CPE) × province mean income (Model 4c). To illustrate these results, the interactions are presented in Figs 1–3. The three economic characteristics were transformed back from log to absolute values for interpretation. Critical values were selected as follows: PCLS at 5, 10, 15, 20, and 25 m²; CEE at 0.018, 0.135, and 1 (10,000 Yuan); and CPE at 0.135, 1, 7.389, and 54.598 (10,000 Yuan).

Fig 1 illustrates the interaction between PCLS and province mean income for BMI. As province mean income increases, the predicted median BMI decreases across all PCLS levels. The slope is steepest for individuals with a PCLS of 25 m², while it is more gradual for those with a PCLS of 5 m². The lines for different PCLS levels converge at higher mean income values, suggesting that the effect of PCLS on BMI diminishes as province income rises. Notably, all lines intersect around a mean income of approximately 36,000 Yuan, after which the differences between groups become more pronounced again. In provinces with lower mean incomes, individuals with a PCLS of 25 m² have the highest median BMI within the normal range, whereas those with a PCLS of 5 m² have the lowest. In provinces where mean income exceeds 36,000 Yuan, this pattern reverses: individuals with a PCLS of 5m² exhibit the highest median BMI within the normal range, while those with a PCLS of 25 m² have the lowest.

Fig 2 shows the interaction between cultural entertainment expenses (CEE) and province mean income for SRH. The lines representing different CEE levels converge at higher province incomes, indicating that the effect of CEE on SRH diminishes as income increases. The lines intersect at approximately 39,000 Yuan, after which the differences between groups again become more pronounced. In provinces with lower mean incomes, individuals with a CEE of 1 (unit: 10,000 Yuan) have the highest probability of reporting good SRH, while those with a CEE of 0.018 have the lowest. This pattern reverses in provinces with mean incomes above 39,000 Yuan: individuals with a CEE of 0.018 report the highest probability of good SRH, whereas those with a CEE of 1 report the lowest.

Fig 3 presents the interaction between car purchase expenses (CPE) and province mean income for SRH. In provinces with lower mean incomes, individuals with a CPE of 54.598 (unit: 10,000 Yuan) have the highest probability of reporting good SRH, while those with a CPE of 0.135 (unit: 10,000 Yuan) have the lowest. The lines converge at approximately 25,000 Yuan, after which the pattern reverses: in higher-income provinces, individuals with a CPE of 54.598 (unit: 10,000 Yuan) exhibit the lowest probability of reporting good SRH, while those with a CPE of 0.135 (unit: 10,000 Yuan) have the highest.

#### Heterogeneous effects by gender.

To test the robustness of our findings and explore potential gender differences, we conducted stratified analyses by gender based on Model 3 (Table 3, Model 3−1). Among females, higher values of durable goods were significantly associated with a decreased likelihood of maintaining a normal BMI (OR = 0.965). Higher tourism expenditures, larger living areas, and greater household cash liquidity were associated with better self-rated health (ORs = 1.041, 1.001, and 1.029, respectively), consistent with the results from the full sample. Among males, higher province mean income and greater values of durable goods were significantly associated with a decreased likelihood of maintaining a normal BMI (ORs = 0.958 and 0.947, respectively). Similarly, higher tourism expenditures, larger living areas, and greater household cash liquidity were associated with better self-rated health (ORs = 1.027, 1.002, and 1.031, respectively), all consistent with the results from the full sample. It is noteworthy that the total government subsidies and total value of durable consumer goods became significant in the gender-stratified models (for both females and males) but not in the full-sample model. This suggests potential gender-specific pathways linking economic resources to health outcomes, consistent with prior evidence that socioeconomic determinants of health may operate differently between men and women in China.

**Table 3:** Multivariate multilevel model results for BMI and self-rated health (odds ratios with 95% confidence intervals): stratified analysis by gender.

	Model 3−1							
	Normal BMI				Good SRH			
	Gender: female		Gender: male		Gender: female		Gender: male	
	ORs	95% CI	ORs	95% CI	ORs	95% CI	ORs	95% CI
**Fixed Part**								
Age	0.995	(0.993–0.997)***	0.997	(0.995–0.999)***	0.967	(0.965–0.969)***	0.969	(0.966–0.97)***
Hukou (ref: urban hukou)								
Rural hukou	0.993	(0.921–1.069)	1.063	(0.985–1.146)	1.08	(0.994–1.174)*	1.149	(1.053–1.254)***
Unknown	0.949	(0.83–1.085)	1.168	(1.02–1.338)**	0.931	(0.803–1.079)	0.969	(0.834–1.129)
Education (ref: No formal educated)								
Primary	0.994	(0.908–1.087)	0.987	(0.892–1.092)	1.031	(0.943–1.13)	1.021	(0.925–1.129)
Middle	1.095	(1.002–1.198)**	0.988	(0.896–1.09)	1.29	(1.176–1.416)***	1.229	(1.113–1.358)***
High school	1.204	(1.08–1.343)***	0.983	(0.88–1.1)	1.672	(1.483–1.883)***	1.426	(1.266–1.606)***
College and above	1.317	(1.16–1.493)***	0.966	(0.848–1.099)	1.818	(1.565–2.115)***	1.564	(1.342–1.824)***
Missing	1.354	(1.087–1.687)***	1.1	(0.889–1.361)	1.107	(0.878–1.395)	0.974	(0.771–1.231)
BMI in the last wave (ref: Abnormal)								
Normal	6.6	(6.178–7.043)***	8.24	(7.706–8.811)***				
New respondents	2.852	(2.627–3.093)***	3.013	(2.784–3.261)***				
Missing information in the last wave	2.718	(2.354–3.136)***	3.165	(2.68–3.736)***				
Self-rated heath in the last wave (ref: others)								
Good					4.591	(4.284–4.918)***	4.568	(4.25–4.914)***
New respondents					3.05	(2.77–3.353)***	3.111	(2.812–3.442)***
Missing information in the last wave					2.401	(1.598–3.611)***	3.766	(1.441–9.836)***
Mean income at province level	0.964	(0.918–1.011)	0.958	(0.913–1.004)*	1.011	(0.937–1.091)	1.036	(0.96–1.119)
Total income from employment	1.001	(0.99–1.012)	0.994	(0.983–1.005)	1.007	(0.995–1.019)	1.004	(0.992–1.017)
Total government subsidies	0.997	(0.974–1.019)	1.007	(0.985–1.029)	0.97	(0.947–0.994)**	0.96	(0.937–0.983)***
Monthly food expenses	1.014	(0.972–1.057)	0.962	(0.922–1.002)*	0.978	(0.936–1.023)	1.002	(0.957–1.049)
Expenses on culture and entertainment	1.02	(0.984–1.058)	1.009	(0.973–1.047)	1.027	(0.986–1.07)	0.994	(0.952–1.038)
Travel expenses	1.006	(0.982–1.03)	0.991	(0.967–1.015)	1.041	(1.013–1.069)***	1.027	(0.998–1.058)*
Expenses on car purchases	0.995	(0.977–1.012)	0.984	(0.968–1.001)*	0.985	(0.966–1.004)	0.999	(0.979–1.019)
Healthcare expenses	1.015	(0.985–1.046)	1.005	(0.974–1.036)	0.983	(0.951–1.017)	0.99	(0.956–1.025)
Per capita living area	1.001	(1–1.003)*	1	(0.999–1.002)	1.001	(1–1.003)*	1.002	(1–1.003)**
Total value of durable consumer goods	0.965	(0.949–0.98)***	0.947	(0.932–0.963)***	1.051	(1.033–1.07)***	1.042	(1.024–1.06)***
Total cash and deposits	1.002	(0.992–1.012)	0.994	(0.983–1.004)	1.029	(1.018–1.042)***	1.031	(1.019–1.044)***
Random Part								
Level4: province								
Variance	0.005		0.005		0.046		0.056	
Correlation	0.004		−0.014		0.004		−0.014	
Level3: Year								
Variance	0.017		0.018		0.01		0.003	
Correlation	−0.009		−0.01		−0.009		−0.01	
Level2: individualss								
Correlation	0.02		0.016		0.02		0.016	

#### Heterogeneous effects by education.

Similarly, to examine whether the associations vary across different educational attainment levels, we conducted subgroup analyses by education level (lower education: no formal or primary education; higher education: middle school and above). (Table 4, Model 3−2). In both groups, higher values of durable goods were significantly associated with a decreased likelihood of maintaining a normal BMI (ORs = 0.950 and 0.961, respectively), but with a higher likelihood of reporting good SRH (ORs = 1.041 and 1.049, respectively). Higher province mean income was also significantly associated with a lower likelihood of maintaining a normal BMI (ORs = 0.941 and 0.953, respectively). In addition, greater tourism expenditures (ORs = 1.051 and 1.042, respectively) and larger household cash liquidity (ORs = 1.039 and 1.025, respectively) were positively associated with better SRH, consistent with the findings from the full sample.

**Table 4:** Multivariate multilevel model results for BMI and self-rated health (odds ratios with 95% confidence intervals): stratified analysis by educational attainment.

	Model 3−2							
	Normal BMI				Good SRH			
	Education: primary school and below		Education: middle school & above		Education: primary school and below		Education: middle school & above	
	ORs	95% CI	ORs	95% CI	ORs	95% CI	ORs	95% CI
**Fixed Part**								
Age	0.997	(0.994–0.999)***	0.996	(0.994–0.998)***	0.972	(0.97–0.975)***	0.962	(0.96–0.964)***
Male (ref: female)	1.154	(1.083–1.229)***	0.824	(0.782–0.869)***	1.428	(1.339–1.522)***	1.288	(1.208–1.372)***
Hukou (ref: urban hukou)								
Rural hukou	1.046	(0.95–1.15)	1.009	(0.947–1.075)	1.009	(0.915–1.112)	1.101	(1.019–1.189)**
Unknown	1.116	(0.961–1.297)	1.004	(0.884–1.14)	0.897	(0.77–1.046)	0.936	(0.803–1.092)
BMI in the last wave (ref: Abnormal)								
Normal	5.463	(5.094–5.859)***	9.384	(8.811–9.994)***				
New respondents	2.45	(2.223–2.699)***	3.37	(3.149–3.607)***				
Missing information in the last wave	2.614	(2.264–3.016)***	3.102	(2.627–3.666)***				
Self-rated heath in the last wave (ref: others)								
Good					4.25	(3.967–4.554)***	4.998	(4.641–5.376)***
New respondents					2.284	(2.067–2.524)***	3.615	(3.297–3.967)***
Missing information in the last wave					2.438	(1.621–3.666)***	2.824	(1.151–6.917)**
Mean income at province level	0.941	(0.892–0.993)**	0.953	(0.912–0.997)**	1.001	(0.917–1.093)	1.066	(0.987–1.151)
Total income from employment	0.995	(0.982–1.008)	0.999	(0.989–1.008)	1.006	(0.992–1.019)	1.01	(0.998–1.021)*
Total government subsidies	1.011	(0.987–1.035)	0.993	(0.971–1.014)	0.96	(0.937–0.982)***	0.969	(0.945–0.993)**
Monthly food expenses	1.057	(1.011–1.103)**	0.931	(0.895–0.969)***	1.009	(0.966–1.054)	0.988	(0.943–1.036)
Expenses on culture and entertainment	1.006	(0.958–1.057)	1.023	(0.992–1.054)	0.997	(0.948–1.048)	1.023	(0.986–1.063)
Travel expenses	0.996	(0.962–1.031)	1	(0.981–1.02)	1.051	(1.015–1.089)***	1.042	(1.017–1.066)***
Expenses on car purchases	0.995	(0.973–1.017)	0.986	(0.971–1.001)*	0.989	(0.968–1.011)	0.99	(0.972–1.008)
Healthcare expenses	1.007	(0.965–1.05)	1.013	(0.988–1.039)	0.994	(0.952–1.038)	0.994	(0.966–1.024)
Per capita living area	1	(0.999–1.002)	1.001	(1–1.002)*	1.001	(0.999–1.003)	1.002	(1–1.003)**
Total value of durable consumer goods	0.95	(0.931–0.97)***	0.961	(0.947–0.974)***	1.041	(1.019–1.064)***	1.049	(1.033–1.067)***
Total cash and deposits	1.002	(0.991–1.014)	0.994	(0.985–1.004)	1.039	(1.026–1.051)***	1.025	(1.014–1.037)***
Random Part								
Level4: province								
Variance	0		0.007		0.06		0.058	
Correlation	0		−0.019		0		−0.019	
Level3: Year								
Variance	0.023		0.016		0.013		0.007	
Correlation	−0.005		−0.01		−0.005		−0.01	
Level2: individual								
Correlation	0.016		0.019		0.016		0.019	

Notes: ***p < 0.01, **p < 0.05, *p < 0.1.

It is noteworthy that total government subsidies became significant in the education-stratified models (for both lower- and higher-education groups) for SRH, although they were not significant in the full-sample model. Furthermore, higher monthly food expenditure was associated with an increased likelihood of maintaining a normal BMI among those with lower education (OR = 1.057), whereas the opposite pattern was observed among those with higher education (OR = 0.931). These findings suggest potential education-specific pathways linking economic resources to health outcomes, consistent with prior evidence that socioeconomic determinants of health may operate differently across educational groups in China.

Taken together, these robustness checks indicate that the protective role of economic status at both the individual and province levels is relatively consistent across gender and education, although the strength and statistical significance of the associations vary.

### 3.3. Geographical differences of health outcomes in China

Beyond the direct and moderating effects of individual, household, and province economic factors, understanding the spatial distribution of health outcomes is critical for interpreting health inequality in China. Uneven distribution of economic development and health resources across provinces can create geographic clustering of BMI and SRH, reflecting the influence of regional socioeconomic environments.

**Fig 4:**
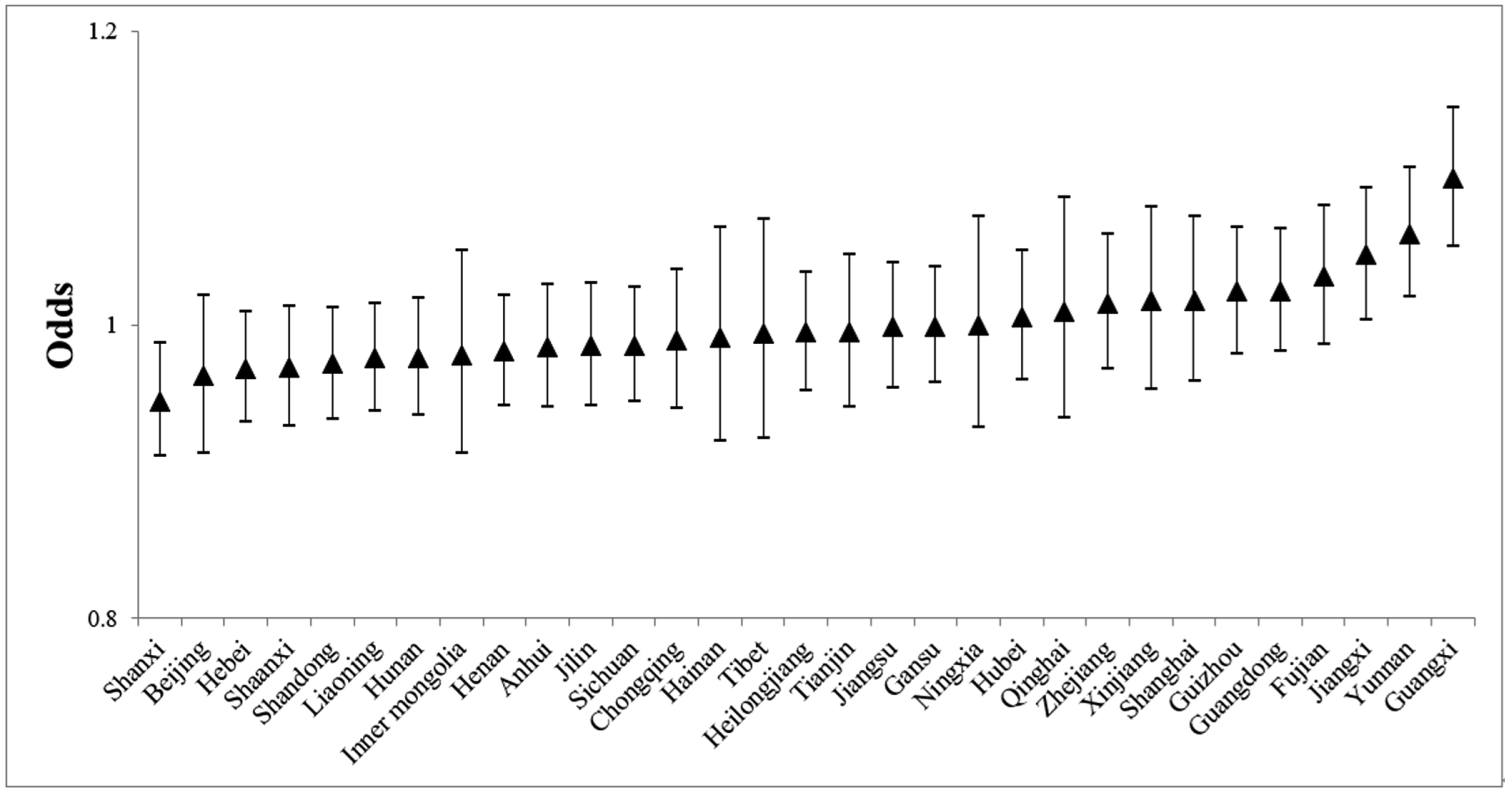
Geographic differentials of BMI.

**Fig 5:**
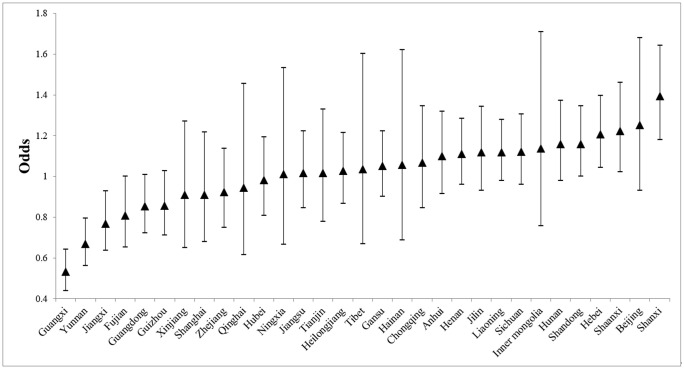
Geographic differentials of SRH.

Table 2 indicates notable between-province variance in both BMI and SRH, which is illustrated in Figs 4 and 5 using level-4 residuals on an odds scale (1 = baseline probability across all provinces). For BMI (Fig 4), southern provinces, including Guangxi, Yunnan, Jiangxi, Fujian, Guangdong, and Guizhou, show higher odds of maintaining a normal BMI, whereas northern provinces such as Shanxi, Beijing, Hebei, Shaanxi, Shandong, and Liaoning have lower odds. Guangxi has the highest odds, while Shanxi has the lowest. Confidence intervals suggest limited intra-regional variation among other provinces. For SRH (Fig 5), a contrasting north–south pattern emerges. Northern provinces, including Shanxi, Beijing, Shaanxi, Shandong, and Hebei, generally report higher odds of good SRH, while southern provinces, including Guangxi, Yunnan, Jiangxi, Fujian, Guangdong, and Guizhou, report lower odds. Shanxi ranks highest in SRH, whereas Guangxi is at the lowest end.The results support Hypothesis 3.

**Fig 6:**
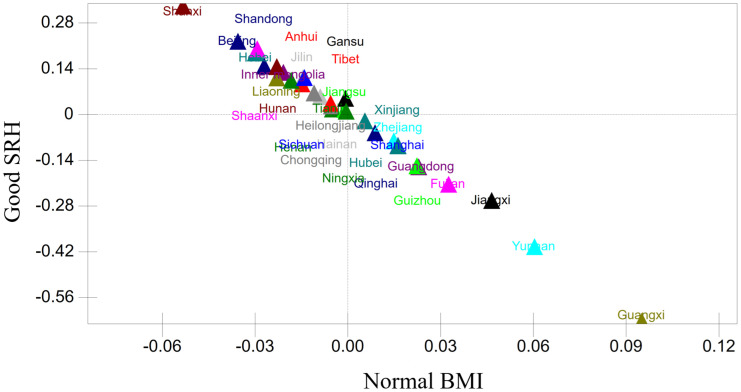
Spatial correlation between BMI and Self-Rated Health (SRH).

Spatial analysis further shows a negative correlation between BMI and SRH across provinces (Table 2; Fig 6). Guangxi, with the highest normative BMI, paradoxically has the lowest SRH, while Shanxi, with the highest SRH, has the lowest normative BMI. This discrepancy highlights the divergence between objective health measures (BMI) and subjective health assessments (SRH), underscoring the role of personal perceptions in shaping self-rated health.

## 4. Discussion and conclusions

This study examined the complex interplay between individual and household economic status, province economic development, and health outcomes, focusing on body mass index (BMI) and self-rated health (SRH). The findings provide several important insights.

Consistent with prior research grounded in health capital theory, the results confirm that micro-level socioeconomic variables exerted a statistically significant influence on health outcomes. Beyond individual income, which most directly reflects individual economic capacity, several consumption-related variables, such as tourism expenditure, housing area, and the total value of durable consumer goods, also significantly associated with health outcomes. Higher tourism expenditure was associated with better SRH, suggesting that individuals who spend more on travel tend to perceive themselves as healthier.

Tourism often involves physical activity, exposure to restorative environments, and stress reduction, thereby promoting both physical and psychological well-being [41]. More investing in leisure activities also showed lower risks of negative emotions and mental health problems, further emphasizing the positive role of recreational consumption in health maintenance [42]. Inside house, per capita living area and the total value of durable consumer goods are two indicators that not only reflect economic standing but also imply living standards. However, these two measures showed opposite associations with optimal BMI. Housing quality has long been recognized as a fundamental determinant of health, linked to stability, indoor environmental conditions, and dwelling size. Neighborhood instability, for instance, has been found to increase risks of cardiovascular disease [43], while poor indoor environmental quality, including exposure to pollutants and lack of ventilation, undermines health [44]. Larger housing areas generally indicate better living environments, greater comfort, and less crowding, all of which enhance psychological well-being and life satisfaction. In contrast, a higher total value of durable consumer goods was associated with a lower likelihood of maintaining an optimal BMI, yet a higher likelihood of reporting good SRH. This may reflect the lifestyle implications of affluence, greater access to high-calorie foods, sedentary habits, and perceived well-being rooted in material satisfaction rather than objective health.

Regarding the absolute income theory, the results demonstrate that province economic development played an important role in shaping both BMI and SRH. Higher levels of province economic development were significantly associated with a lower likelihood of having a normal BMI but a higher likelihood of reporting good SRH. However, the association with SRH became insignificant after controlling for individual or household economic status. These findings partly supported the theory that as economic resources increase, individuals and communities can better afford health-promoting goods and services, leading to overall improvements in population health [27,28]. At the same time, they suggest that higher levels of province economic development may act as a double-edged sword: while promoting better self-rated health, they may also increase the risk of abnormal BMI due to behavioral and lifestyle changes associated with affluence. In addition, province economic development moderated the associations between individual economic resources and health outcomes. Increases in province mean income tend to reduce health disparities linked to per capita living area, cultural and entertainment expenditure, and car purchase expenses, suggesting convergence in BMI and SRH outcomes at higher levels of province economic development. However, these effects were not uniform. Beyond certain province mean income thresholds (approximately 36,000–39,000 Yuan), the patterns reversed, and differences between groups became more pronounced again. Together, these findings highlight that province economic growth not only directly shaped health outcomes but also interacted with household-level economic behaviors, amplifying or mitigating health inequalities depending on the stage of economic development.

Substantial spatial differences in health outcomes were observed, revealing an intriguing inverse relationship between BMI and SRH at the province level. Provinces with higher average BMI tended to less likely to report good SRH, highlighting a divergence between objective and subjective health indicators. This pattern suggests that while BMI reflects physical health risks, SRH captures broader cultural, psychosocial, and environmental dimensions of well-being.

At the province level, the negative association between average BMI and SRH likely reflects structural and environmental dynamics rather than individual perceptions. Provinces with higher mean of normal BMI tend to be more economically developed and urbanized, where sedentary lifestyles, calorie-dense diets, and work-related stress contribute to poorer perceived health despite greater material affluence. In contrast, provinces with lower level of normal BMI often have less developed economies and lower caloric intake but benefit from more active lifestyles, stronger community ties, and lower psychological stress, all of which may enhance self-rated health [45]. Moreover, regional disparities in healthcare access [46,47], dietary traditions (e.g., oil-heavy cuisines in northern regions versus lighter diets in the south) [5], and environmental quality (e.g., air pollution, food safety, and urban green space) may further mediate this relationship. Collectively, these findings underscore how China’s rapid socioeconomic transition has produced a “dual health burden,” with obesity-related conditions in affluent provinces coexisting alongside undernutrition and limited healthcare access in less developed regions.

At the individual level, interpersonal variations in health outcomes may reflect culturally embedded body ideals. The “thin-is-beautiful” norm may lead some individuals to associate lower level of normal BMI with better health, whereas others with abnormal BMI may still report good SRH due to body acceptance, social comparison, or local dietary norms. The Chinese saying “a contented mind, a portly frame” encapsulates this cultural nuance, suggesting that subjective well-being may, at times, outweigh objective physical indicators in shaping health perceptions.

This study thus bridges micro- and macro-level determinants of health by demonstrating that household economic resources and province economic development jointly shape individual health outcomes. Individual and household socioeconomic variables remain strong predictors of BMI and SRH, but their effects were conditioned by the broader province context. Moreover, distinct regional disparities persist, with BMI and SRH showing an inverse relationship across provinces.

These findings have several policy implications. Promoting health equity requires coordinated interventions across multiple levels. At the individual- or household- level, improving economic capacity through targeted poverty alleviation, social welfare, and education can directly enhance health. The positive association between tourism expenditure and SRH suggests that encouraging participation in recreational and cultural activities may improve both physical and mental well-being. Likewise, expanding programs that enhance housing quality and affordability can contribute measurable health benefits.

At the province level, the results highlight a paradox: southern provinces display healthier BMI distributions yet were less likely to report good SRH, reflecting a misalignment between objective and subjective health. Addressing this gap calls for province-specific strategies such as health education, chronic disease prevention programs, and improvements in healthcare access and quality. Reducing interprovince disparities in health infrastructure is crucial to narrowing regional health gaps. Public health investments should be tailored to local socioeconomic and cultural contexts, recognizing that province income interacts with household resources to shape health inequalities. Integrating these approaches into ongoing health reforms and the "*Healthy China 2030*" strategy can help align perceived and objective health outcomes, fostering both equity and well-being.

## Supporting information

S1 File Data. (XLSX)
